# Supplementary material for: Glioma Stem Cells Are Sensitized to BCL-2 Family Inhibition by Compromising Histone Deacetylases
Source: Int J Mol Sci. 2023 Sep 5;24(18):13688. doi: 10.3390/ijms241813688 (PMC10530722; doi:10.3390/ijms241813688)
Supplement: Supplementary file 1 [file ijms-24-13688-s001.zip › ijms-2558267-supplementary.pdf]

Figure S1

Gene Mutations	U87	LN229	PDX 12*	PDX 39*	PDX 14*
TERT	**C228T (-124C>T) TERT promoter mutant (PMID=31068700).**	C2280T (-124C>T) TERT promoter mutant (PMID=31068700).	C250T (-146C>T) *TERT* promoter mutant	C250T (-146C>T) *TERT* promoter mutant	C228T (-124C>T) TERT promoter mutant
**RAS pathway**	**Heterozygous for NF1 p.Phe1247fs*18 (c.3737_3740delTGTT) (CCLE; Cosmic-CLP).**	**None detected**	None detected	None detected	None detected
EGFR	**WT (PMID: 24135280)**	**WT (PMID: 24135280)**	WT, amplified	VIII	WT, gain
MGMT	**Negative/deficient (PMID: 27423571)**	**Negative/deficient ((PMID: 29164620)**	methylated/deficient	methylated/deficient	unmethylated
IDH1* and *IDH2	WT (PMID: 32208352)	WT (PMID: 31242696)	WT	WT	WT
**TP53 Status**	Has no TP53 mutation	Homozygous for TP53 p.Pro98Leu (c.293C>T) (ClinVar=VCV000528236) PubMed=10416987	None detected	None detected	None detected
PTEN	Homozygous for PTEN c.209+1G>T; splice donor mutation (CCLE; Cosmic-CLP).	None detected	None detected	None detected	None detected
LIFR	None detected	Heterozygous for LIFR p.Pro1060Ala (c.3178C>G) (Cosmic-CLP)	None detected	None detected	None detected
RAD21	None detected	Heterozygous for RAD21 p.Gln132Ter (c.394C>T) (Cosmic-CLP)	None detected	None detected	None detected

Figure S1. PDX and cell cultured adapted GBM lines used in this study. Data for U87 and LN229 are from Cellosauraus

Data for GSCs are from the Mayo Clinic:
*https://www.mayo.edu/research/labs/translational-neuro-oncology/mayo-clinic-brain-tumor-patient-derived-xenograft-national-resource/pdx-characteristics/pdx-phenotype

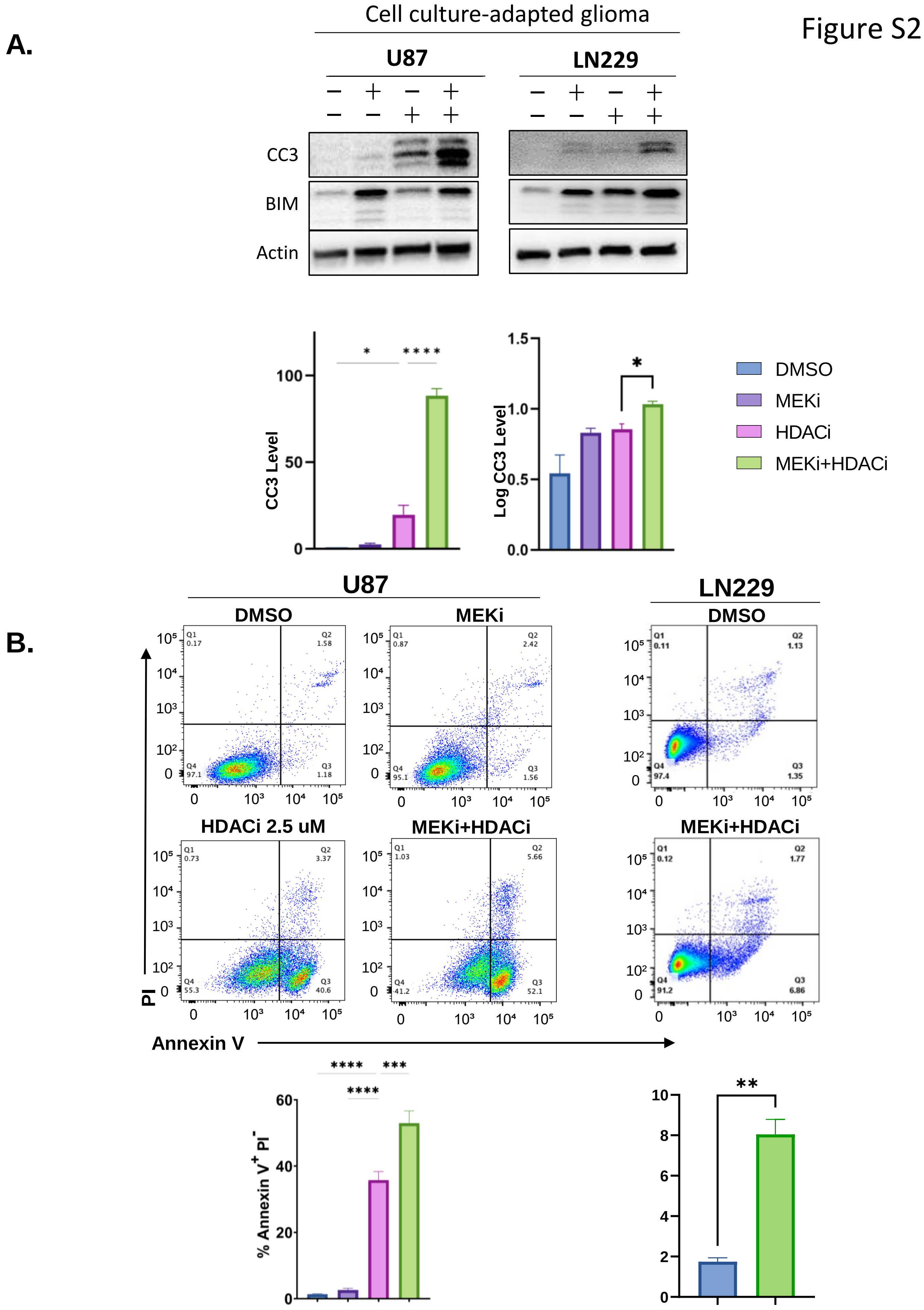

Figure S2. Combining MEKi and HDACi induces apoptosis in glioma stem cells and cell cultured-adapted glioblastoma. A. Western blot for apoptotic marker cleaved caspase-3 (CC3) in U87 and LN229 cells. Cells were treated for 24 h. U87 were treated with HDACi (2.5 µM vorinostat) and MEKi (25 nM trametinib) while LN229 cells were treated with 75 nM trametinib; β-Actin protein was used as a loading control and for normalization. The graphs quantify CC3 expression levels. Data are means +/- SEM (n=3). **B**. Annexin V-propidium iodide (PI) flow cytometry measuring apoptosis (Annexin V positive/PI negative, Q3) for cells treated as in A. Graphs portray the mean + SEM for the percentage of Annexin V+/PI- cells from three independent experiments. p = *≤0.05; **≤0.01 ***≤0.001 ****≤0.0001, t-test.

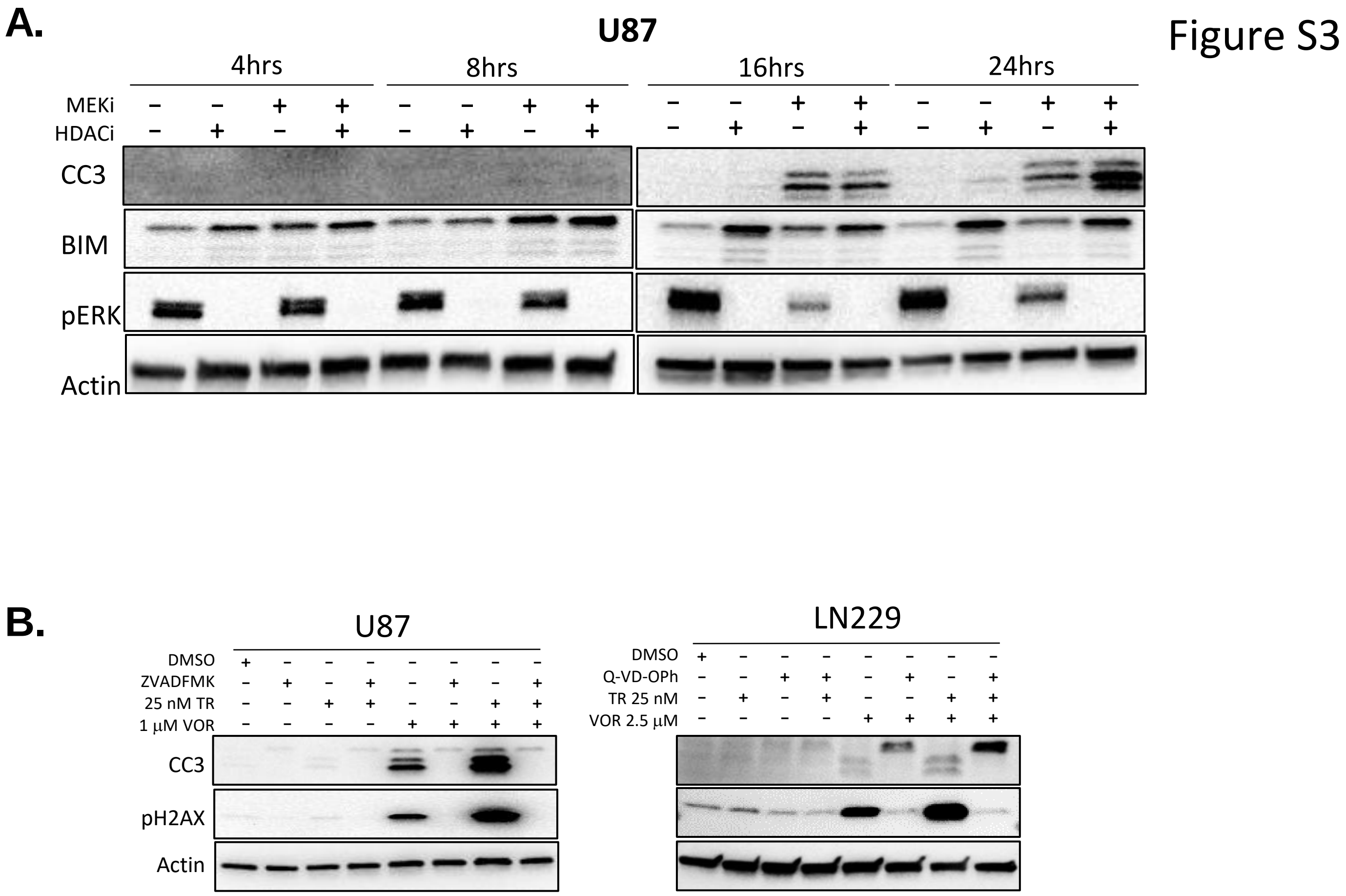

Figure S3. Time course of CC3 and pH2AX after MEKi+HDACi. A time course of U87 cells following 24 h incubation with 25 nM TR and/or 2.5 μM VOR. **B.** Addition of MEKi + HDACi with or without 50 mM Z-VAD-FMK or Q-VD-OPh.

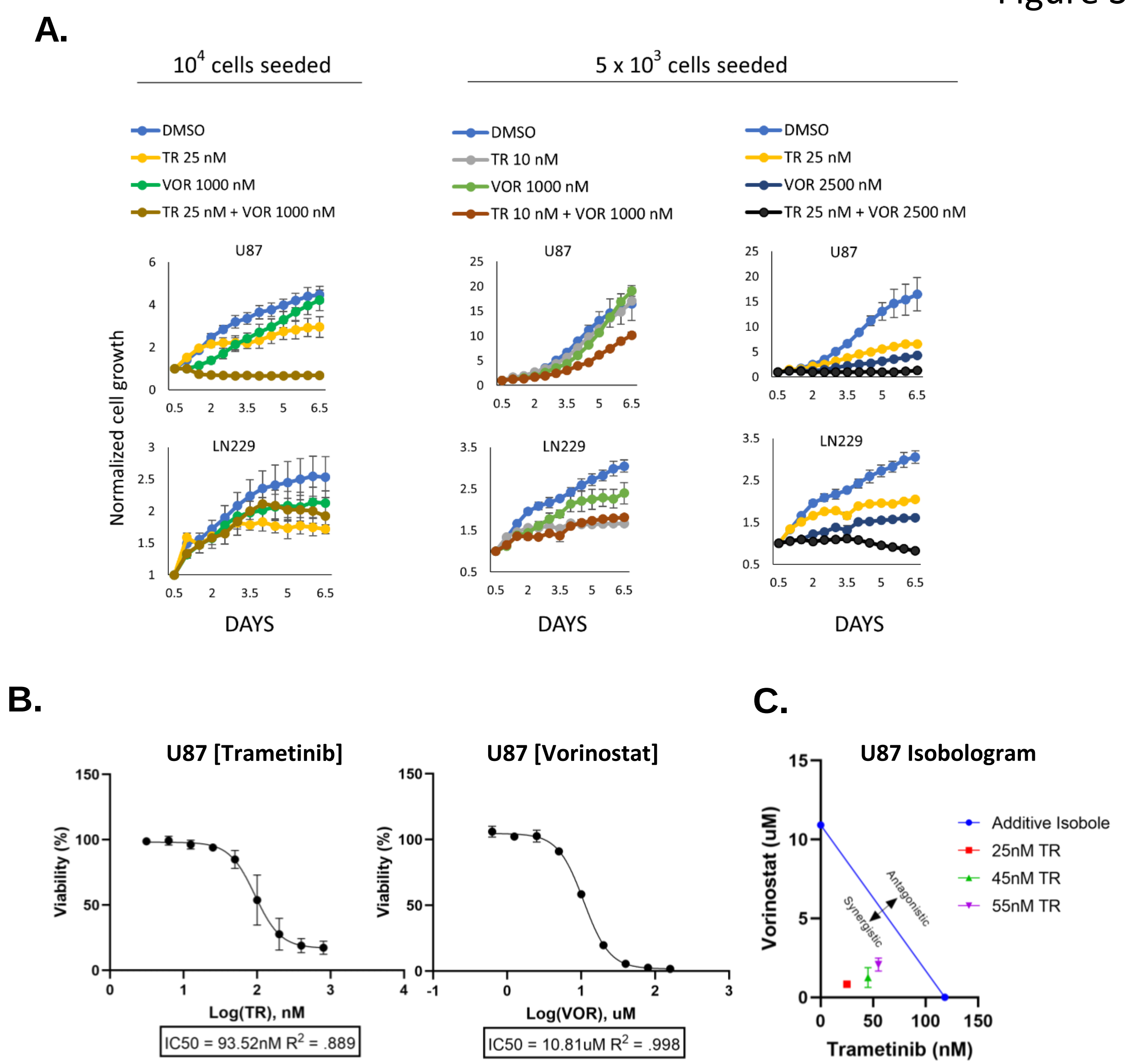

Figure S4. Combination MEKi + HDACi severely impairs growth and viability of GBM cells. A. 10^4 or 5 x 10^3 cells were seeded in 96 well plates and after 12 hours treated with vehicle, or trametinib (TR), vorinostat (VOR) or both followed by automated cell counting using a BioSpa-equipped Cytation 5 for 6.5 days. **B**. Cytotoxicity curves for U87 glioblastoma cells (1,000/well) following 3-day drug incubation with either MEKi by trametinib (TR) or HDACi by vorinostat (VOR). Cell viability measured by luminescence using CellTiterGlo® and normalized to controls such that no drug wells equate to 100% viability. Data are means and SEM (n=3). **C**. Isobologram for MEKi (TR) and HDACi (VOR) in U87s at multiple concentrations below the IC50 of TR alone in combination with VOR. Cell viability measured by luminescence using CellTiterGlo® and normalized to controls such that no drug wells equate to 100% viability. Isobole values recorded based on the first measured concentration yielding 50% viability (IC50). Data are means + SEM (n=3). T = trametinib.

Figure S5

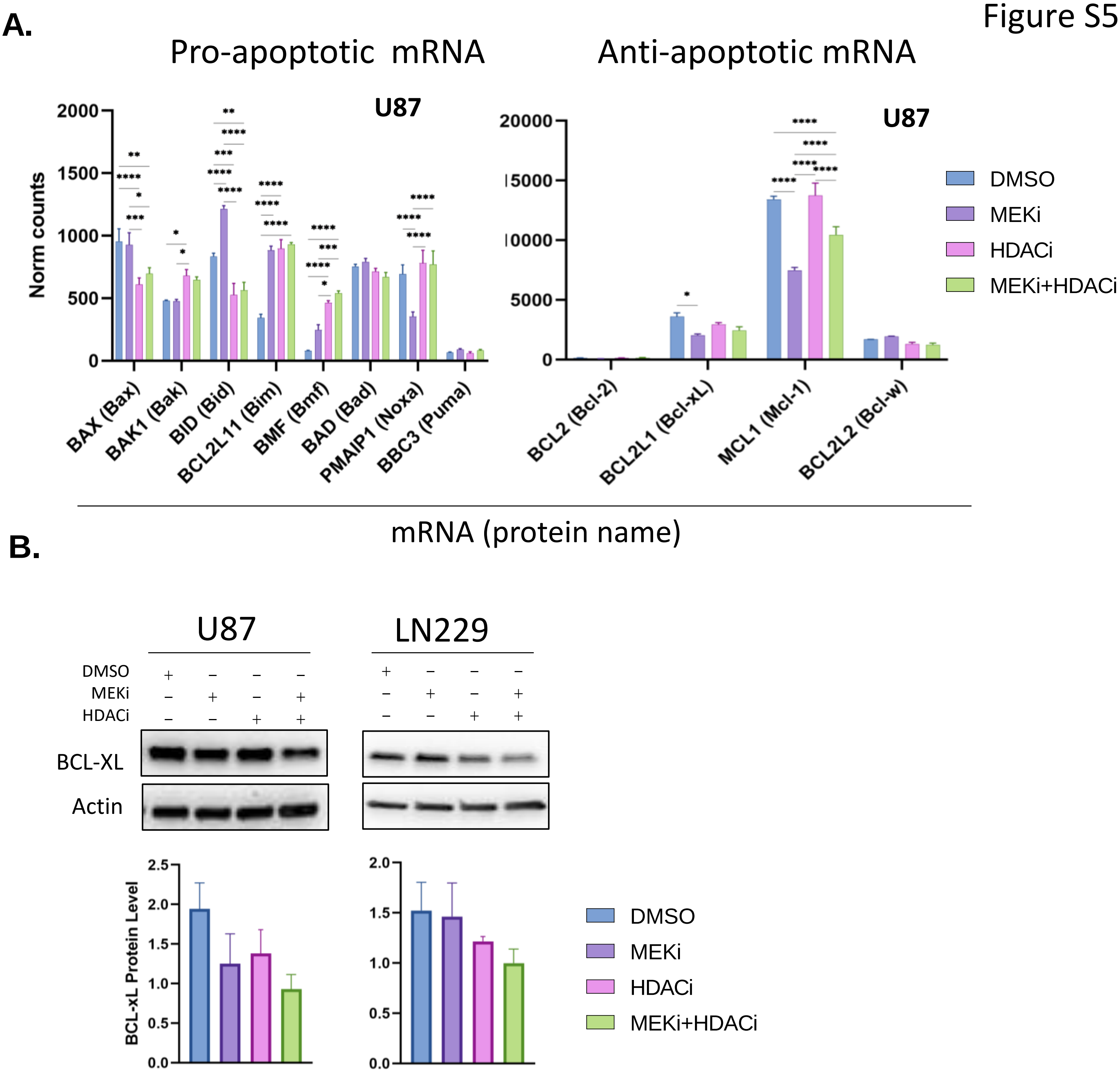

Figure S5. MEKi+HDACi significantly alters expression of major pro- and anti-apoptotic factors. A. RNA-sequencing data for pro-apoptotic (left) and anti-apoptotic (right) genes in U87 cells following 24 h incubation with 25 nM TR and/or 2.5 µM VOR. Data are means and SEM from three independent experiments. Data are means and SEM (n=3). Significance was tested using a multiple comparison one-way ANOVA *≤0.05; **≤0.01 ***≤0.001 ****≤0.0001. Multiple comparisons were Tukey's test. **B.** Anti-apoptotic BCL-xL protein expression in U87 (n = 3) and LN229 (n = 2) cells treated as in A. Data are means + SEM.

Figure S6

A.

BIM/BCL2L11 protein

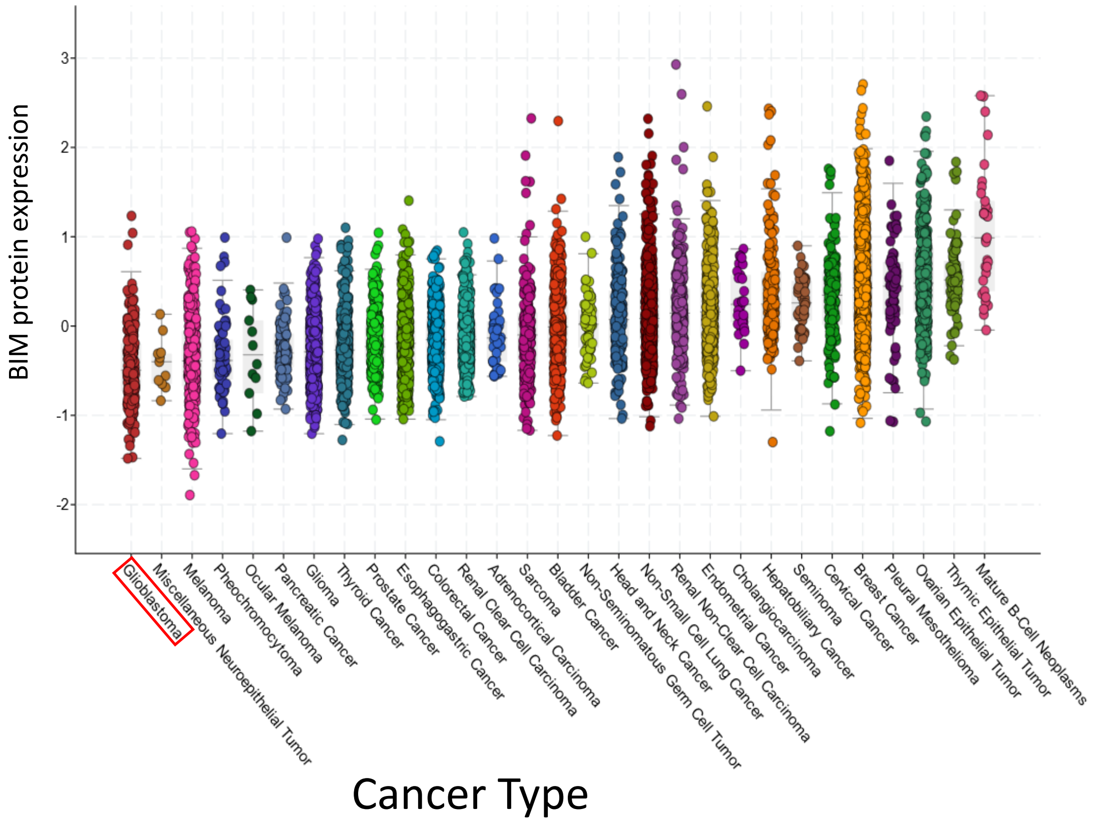

B.

BIM/BCL2L11 mRNA

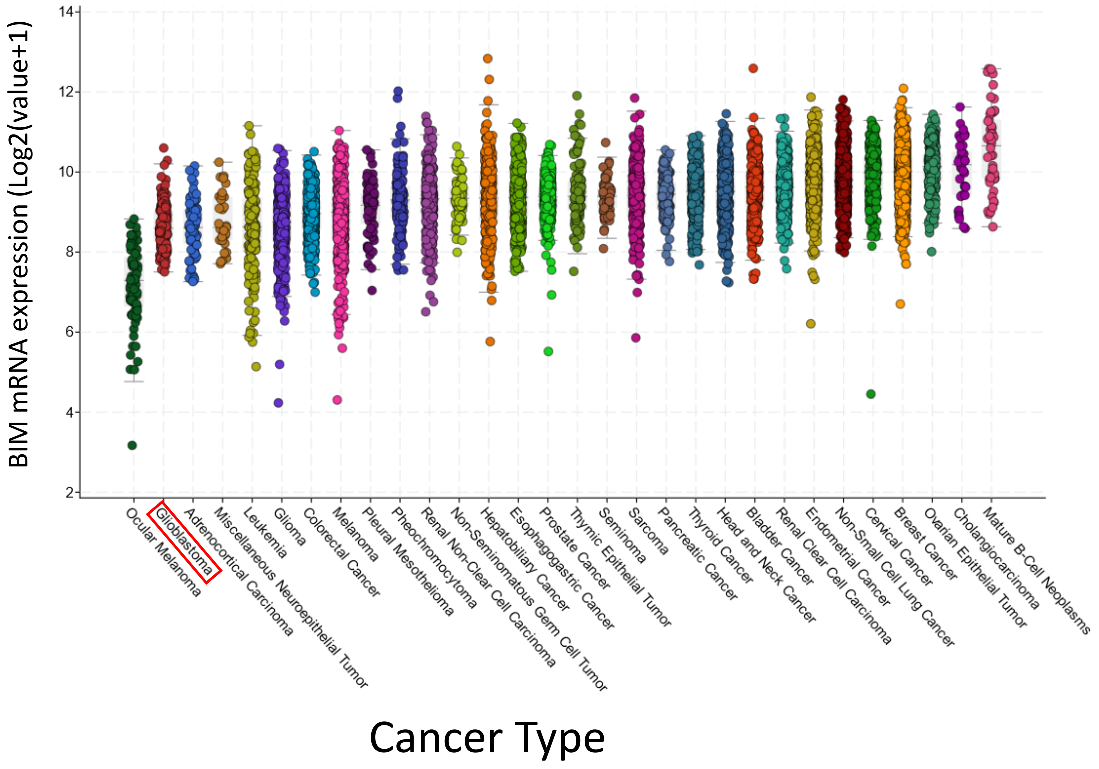

Figure S6. **Relatively low expression of BIM in patient GBM tumors. A.** BIM/BCL2L11 protein and **B.** mRNA expression in patient tumors. Data captured from TCGA cBioBortal. Cancer types are arranged by expression level.

Figure S7

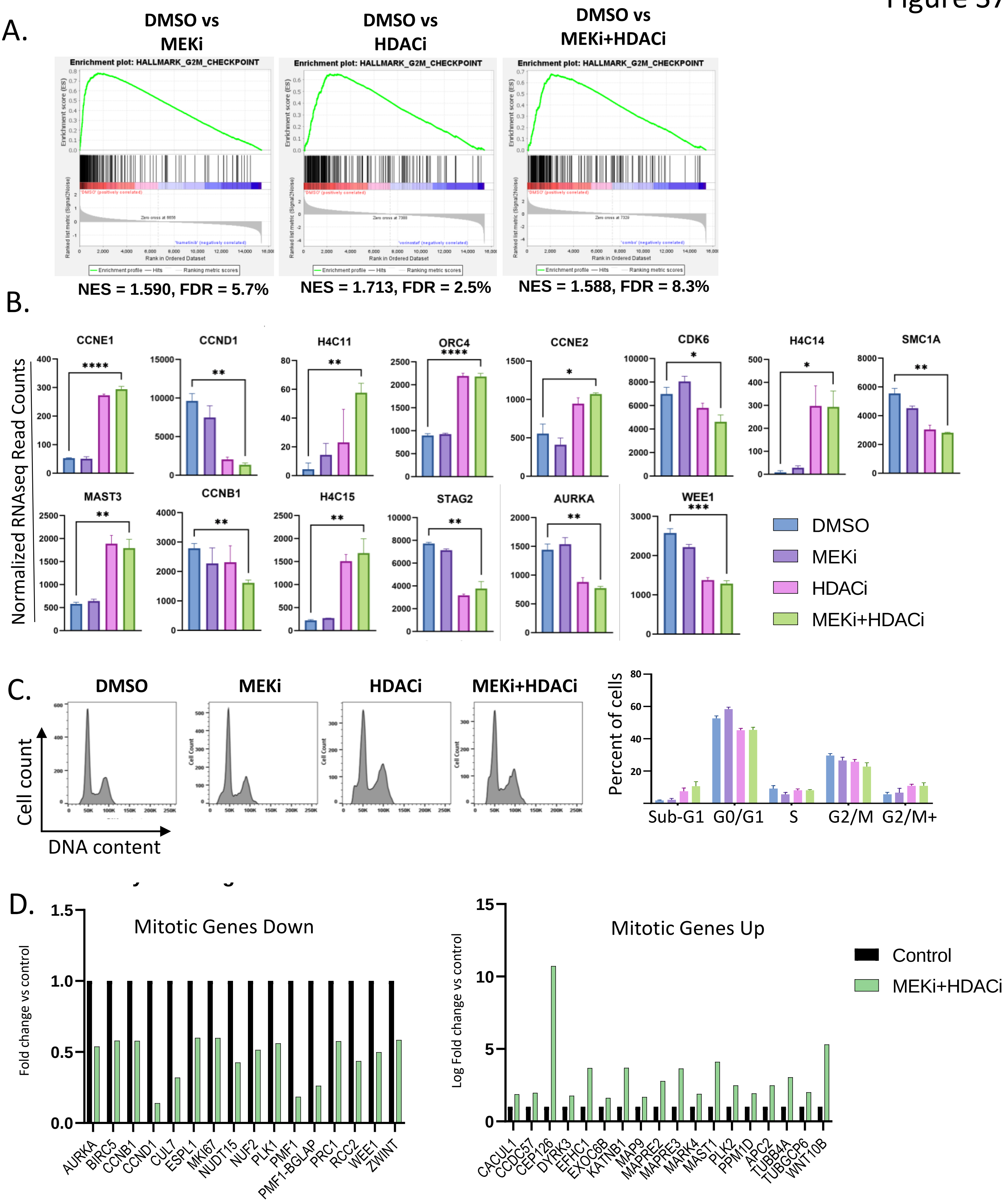

Figure S7. MEKi+HDACi treatment of GSCs altered S phase and mitotic cell cycle factors. A. Hallmark gene expression analysis for G2/M gene expression changes (NES, normalized enrichment score; FDR, false discovery rate) and normalized RNA-sequencing counts for selected cell cycle genes in GSC12 glioblastoma cells following 24 h incubation with 25 nM MEKi and/or 2.5 µM HDACi. Data are means + SEM (n=3). p = *≤0.05; **≤0.01 ***≤0.001 ****≤0.0001, t-test. **B.** mRNA levels of key cell cycle genes after 24 h treatment with MEKi+HDACi in GSC12 cells. Data are means + SEM from 3 independent replicates. **C.** Propidium iodide (PI) flow cytometry analysis of cell cycle for cells treated with MEKi [25 nM trametinib (TR)] and/or HDACi [2.5 µM vorinostat]. Graphs portray the mean + SEM for the percentage of cells in G1, S, or G2/M phases from three independent experiments. **D.** mRNA levels of key mitotic genes after 24 h treatment with MEKi+HDACi in GSC12 cells. Data are means from 3 independent replicates.

Figure S8

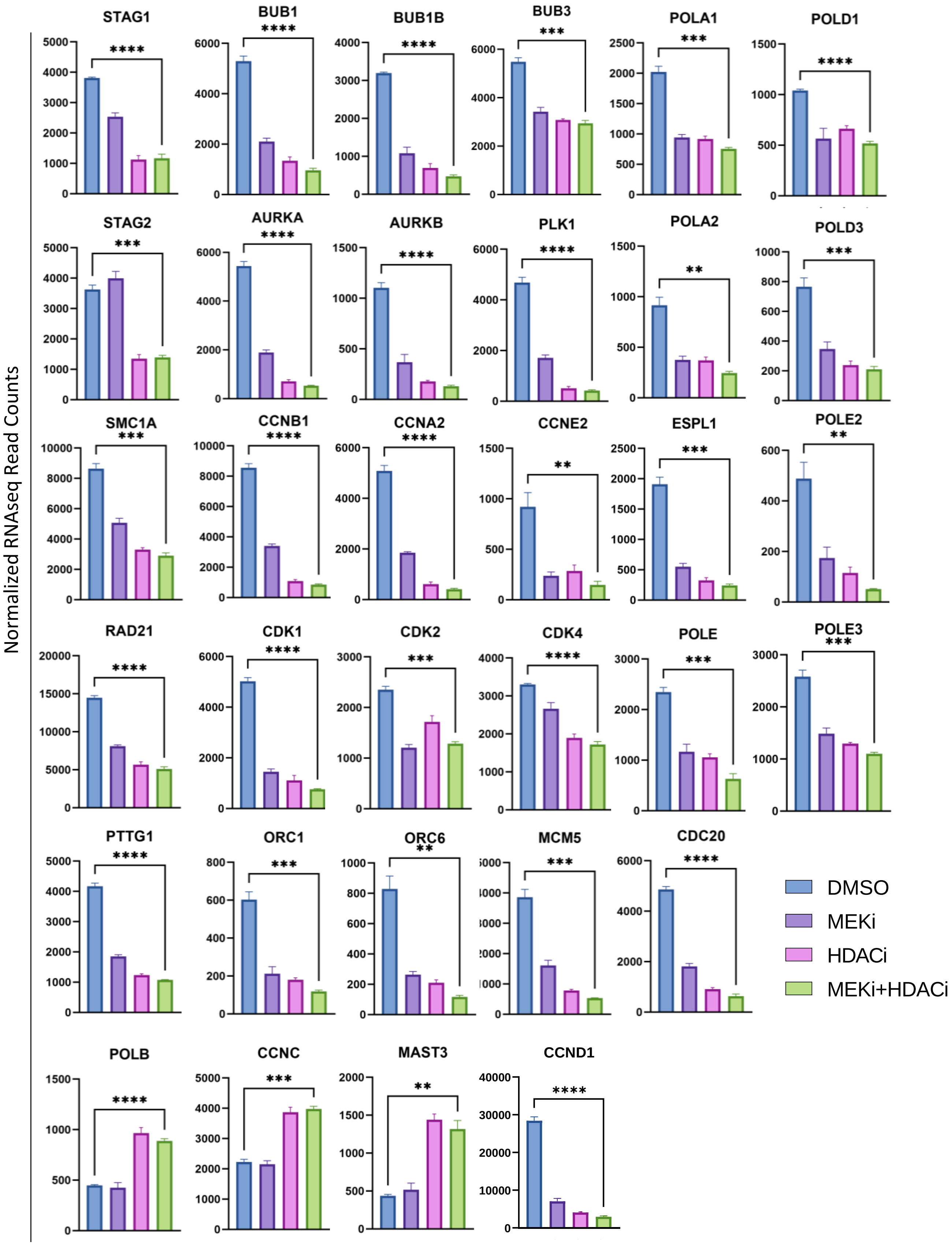

Figure S8. Combinatorial effects of MEKi + HDACi on expression of select cell cycle gene expression in U87 cells. Normalized RNA-sequencing counts for cell cycle genes in U87 glioblastoma cells following 24 h incubation with 25 nM MEKi and/or 2.5 µM HDACi. Data are means + SEM (n=3). p = *≤0.05; **≤0.01 ***≤0.001 ****≤0.0001, t-test.

Figure S9

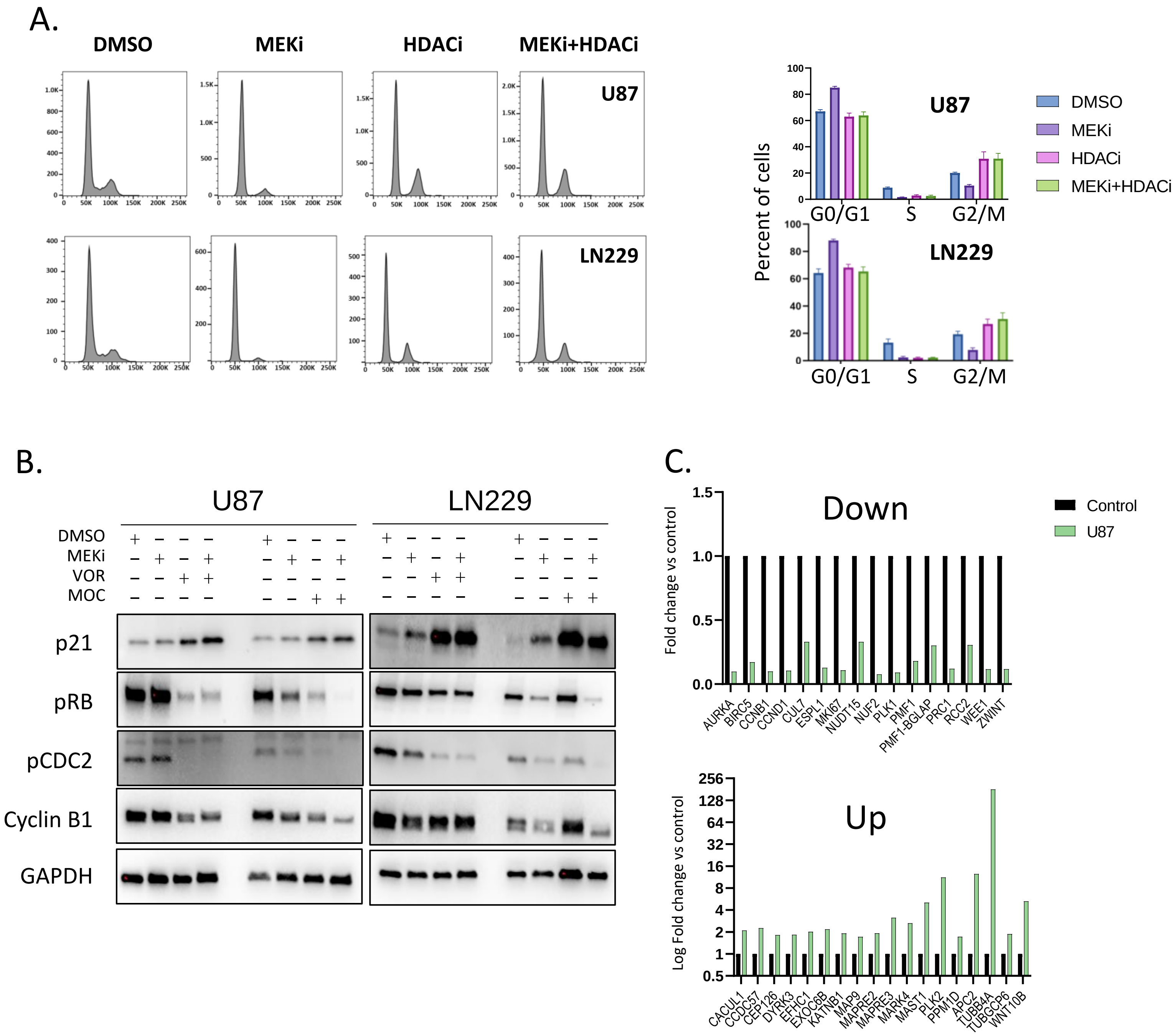

Figure S9. MEKi+HDACi treatment of GBM cells altered S phase and mitotic cell cycle factors. A. Propidium iodide (PI) flow cytometry analysis of cell cycle for cells treated with MEKi [25 nM trametinib (TR)] and/or HDACi [2.5 μM vorinostat]. Graphs portray the mean + SEM for the percentage of cells in G1, S, or G2/M phases from three independent experiments. **B**. Western blot for cell cycle markers in U87 and LN229 cells treated with MEKi and/or HDACi [2.5 μM vorinostat] or 1 μM mocetinostat. Cells were treated for 24 h. GAPDH protein was used as a loading control and for normalization. Phosphorylation of the RB protein (pRB) is a critical regulatory step in allowing cell cycle progression from G1 into S phase. p21 acts an inhibitor of cell cycle progression. Cyclin B1 promotes G2/M phase progression and is degraded at the end of mitosis. Phosphorylation of CDC2 is required for G2/M phase progression. **C.** mRNA levels of key mitotic genes after 24 h treatment with MEKi+HDACi in U87 cells. Data are means from 3 independent replicates.

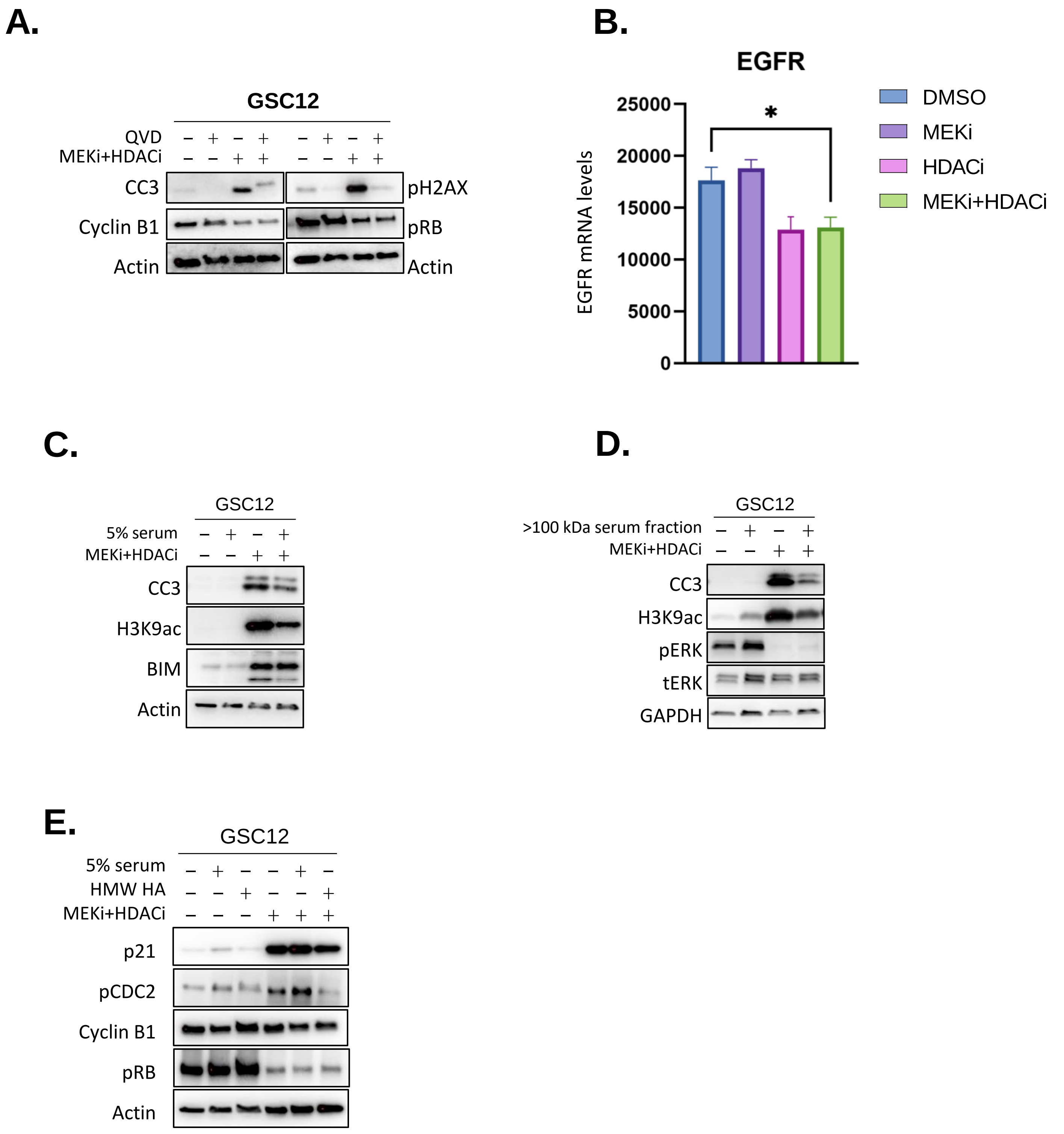

Figure S10. GSC apoptosis induced by MEKi + HDACi does not arise from DNA damage but associates with repressed EGFR mRNA and is rescued by serum factors. A. Cell cycle protein expression after MEKi+HDACi with or without blocking apoptosis/caspase activation using 50 μM Q-VD-OPh. The left and right images are from separate immunoblots of the sample samples. **B.** EGFR mRNA expression from RNAseq. Data are means + SEM (n = 3). p = *≤0.05, t-test. **C.** GSC12 cells were treated with MEKi+HDACi (25 nM trametinib, 2.5 μM vorinostat) for 24 h with or without 5% serum followed by analysis of apoptosis (CC3), histone acetylation (H3K9ac) and the pro-apoptotic BIM. Actin protein was used as a loading control. **D.** GSC12 cells were drug treated as in A with or without serum fractionated for >100 kDa components and added at 5%. CC3, histone acetylation, ERK activated (pERK), total ERK (tERK) were monitored for changes. **E.** Representative data for cells treated with MEKi+HDACi for 24 h with or without 5% serum or HMW HA and immunoblotted for cell cycle factors. Data are from the same membrane as shown in Fig 3C.
